# The Impact of TRPV1 on Cancer Pathogenesis and Therapy: A Systematic Review

**DOI:** 10.7150/ijbs.59918

**Published:** 2021-05-11

**Authors:** Li Li, Cheng Chen, Chengyao Chiang, Tian Xiao, Yangchao Chen, Yongxiang Zhao, Duo Zheng

**Affiliations:** 1 Guangdong Provincial Key Laboratory of Regional Immunity and Diseases, Shenzhen University International Cancer Center, Department of Cell Biology and Genetics, School of Medicine, College of Life Sciences and Oceanography, Shenzhen University, Shenzhen 518055, China.; 2 School of Biomedical Sciences, Faculty of Medicine, The Chinese University of Hong Kong, Shatin, NT, Hong Kong.; 3 National Center for International Research of Biological Targeting Diagnosis and Therapy (Guangxi Key Laboratory of Biological Targeting Diagnosis and Therapy Research), Guangxi Medical University, Nanning, China.

**Keywords:** TRPV1, proliferation, cell death, metastasis, therapy, microenvironment.

## Abstract

The transient receptor potential cation channel subfamily V member 1 (TRPV1) is a transmembrane protein that can be activated by various physical and chemical stimuli and is associated with pain transduction. In recent years, TRPV1 was discovered to play essential roles in cancer tumorigenesis and development, as TRPV1 expression levels are altered in numerous cancer cell types. Several investigations have discovered direct associations between TRPV1 and cancer cell proliferation, cell death, and metastasis. Furthermore, about two dozen TRPV1 agonists/antagonists are under clinical trial, as TRPV1 is a potential drug target for treating various diseases. Hence, more researchers are focusing on the effects of TRPV1 agonists or antagonists on cancer tumorigenesis and development. However, both agonists and antagonists may reveal anti-cancer effects, and the effect may function via or be independent of TRPV1. In this review, we provide an overview of the impact of TRPV1 on cancer cell proliferation, cell death, and metastasis, as well as on cancer therapy and the tumor microenvironment, and consider the implications of using TRPV1 agonists and antagonists for future research and potential therapeutic approaches.

## Introduction

Cancer is increasingly recognized as a serious public health concern worldwide; however, the mechanisms of tumorigenesis and development are complex and not well understood. The transformation from normal cells to cancer cells is caused by numerous alterations in various signaling pathways, which lead to enhanced cell proliferation, inhibition of programmed cell death, migration, and invasion.

Many proteins in cancer cells exhibit enhanced or reduced expression levels compared with the levels in normal cells, and these proteins play important roles in tumorigenesis and development. The recently identified transient receptor potential (TRP) channels are subject to expression changes in cancer cells 1, 2. So far, the most studied members of the family have been TRPC1, TRPV6, TRPM1, and TRPM8 3. However, in the previous two decades, researchers have shown an increased interest in TRP subfamily V member 1 (V1), as it appears to play multiple roles in cancer 4, 5. Alterations in both the expression and activity of TRPV1 are associated with tumorigenesis and therapy. The activation of the cation channel TRPV1 by heat and small molecules permeabilizes cells to allow Ca^2+^ and Na^+^ influx 6. A considerable amount of literature has been published on the effects of TRPV1 agonists and antagonists on tumorigenesis and development.

In this review, we summarize the observations reported to date of the changes in TRPV1 expression and channel activity associated with cancers and the effects of these alterations on cancer cell proliferation, death, and metastasis, and the tumor microenvironment. We further discuss the therapeutic potential of targeting TRPV1 in cancer cells. To comprehensively understand the effects of TRPV1 activation on proliferation, cell death, metastasis, and therapy, we provide tables detailing the investigations reviewed so far regarding relevant drug applications, drug concentrations, tissue/cell types, and their effects on the treatment of tumors.

## What is TRPV1

The human TRPV1 protein is encoded by the *TRPV1* gene located on chromosome 17p13. TRPV1 belongs to the transient receptor potential channel vanilloid subfamily and is also known as the capsaicin receptor and vanilloid receptor 1 (VR1). TRPV1 predominantly forms a homotetramer, with each subunit consisting of six transmembrane segments, a pore-forming loop between the fifth and sixth transmembrane domains, and cytoplasmic C- and N-terminal domains 7-10. Initially, TRPV1 was found to be expressed prominently in small-to-medium-sized neurons of the dorsal root, trigeminal, and vagal ganglia 8, 11, and was discovered to be involved in pain transduction 12. Later, TRPV1 expression was reported in non-neuronal system cells, such as arteriolar smooth muscle cells 13, 14 and the bladder urothelium 15. TRPV1 is a nonselective cation channel that can be activated by different physical and chemical stimuli, including temperatures over 43°C, acidic conditions (pH <6), and vanilloids 8, 9, 16. The activation of TRPV1 induces the cellular influx of Ca^2+^ and Na^+^ ions 17-19, and the excess intracellular Ca^2+^ and Na^+^ leads to cell death 20. Numerous putative endogenous and exogenous agonists and antagonists of TRPV1 have been identified and summarized (Table 1). Because TRPV1 is a promising therapeutic target in many human diseases and conditions 21, 22, about two dozen TRPV1 agonists/antagonists are being used in clinical trials, many of which are concerned with pain and inflammation 23, 24.

## Expression of TRPV1 in cancers

TRPV1 expression has been reported to be higher in human primary brain tumors than tumor-free brains. Furthermore, its expression positively correlates with the grading of tumors 64. Compared with in a normal pancreas, TRPV1 mRNA expression is significantly upregulated in human pancreatic cancer and chronic pancreatitis 65. Elevated TRPV1 expression has also been verified in squamous cell carcinoma of the human tongue, prostate carcinoma, and breast cancer 66-68. All the above published work indicated that TRPV1 expression is upregulated in cancers.

## TRPV1 regulates proliferation

TRPV1 channel activation increases the intracellular Ca^2+^ concentration, and Ca^2+^ signaling plays an essential role in cancer cell proliferation, regulation, and survival 69. A few studies have investigated the associations between changes in TRPV1 protein expression and the regulation of cancer cell proliferation. The majority of studies focused on the effects of TRPV1 agonists or antagonists on cell proliferation; however, some of these chemicals regulate proliferation independently of TRPV1 because they either play roles in cells without TRPV1 expression or affect other receptors. In this section, we describe the roles and underlying mechanisms of the *TRPV1* gene and the effects of TRPV1 agonists and antagonists, both independently of and dependent on TRPV1 and other receptors, on proliferation (Table 2).

### TRPV1 expression suppresses cell proliferation

The overexpression of TRPV1 in intestinal epithelial HCT116 cells suppressed the phosphorylation of epidermal growth factor receptor (EGFR) at Y1068, a receptor that is associated with a variety of pro-proliferative signaling pathways 70. According to the results of *in vitro* studies, the depletion of *Trpv1* in mice led to the constitutive phosphorylation of EGFR at Y1068 and subsequently increased the expression of the oncogenes* c-Fos* and* c-Myc*, indicating that TRPV1 is a negative regulator in intestinal tumorigenesis 70. The proliferation of human melanoma A2058 and A375 cells was inhibited after TRPV1 overexpression, as the overexpression induced apoptosis via the calcineurin-ATF3-p53 pathway in these cells 71. It was reported that TRPV1 overexpression prevented the proliferation of human pancreatic cancer PANC-1 cells and human skin carcinoma A431 cells by promoting EGFR ubiquitination and degradation 72, 73. The evidence presented thus far supports the hypothesis that TRPV1 acts to suppress cancer cell proliferation.

### TRPV1 agonist capsaicin affects cell proliferation dependent on TRPV1

Ligand-induced autophosphorylation of the EGFR results in phospholipase C (PLC) activation, which cleaves phosphatidylinositol-4,5-bisphosphate (PIP_2_) into diacylglycerol (DAG) and inositol triphosphate (IP_3_). Then IP3 triggers TRPV1 and Ca^2+^ influx, which activates calpain and subsequently protein tyrosine phosphatase 1B (PTP1B). PTP1B then dephosphorylates EGFR to inhibit intestinal epithelial HCT116 cell proliferation 70. Amantini et al. found that the capsaicin-induced arrest of the cell cycle in the G0/G1 phase and apoptosis mediated by the ATM-p53 and ATM-Fas/CD95 pathways inhibited the proliferation of human urothelial cancer RT4 cells but not that of TCCSUP, J82, or EJ urothelial cancer cell lines, as TRPV1 showed high expression levels in RT4 cells 74. Furthermore, this antiproliferation effect was completely rescued by capsazepine and SB366791, two antagonists of TRPV1 74. Another study illustrated the capsaicin suppression of human renal carcinoma 786-O cell proliferation, which was also reversed by capsazepine treatment 75. Additionally, capsaicin induced apoptosis by activating the p38 and JNK/MAPK pathways, suppressing proliferation 75. However, the opposite effect of capsaicin on proliferation has also been reported. For example, Malagarie-Cazenave et al. discovered that capsaicin promoted human prostate tumor androgen-responsive LNCaP cell growth by activating the PI3K and p44/42 MAPK pathways to suppress ceramide production as well as increasing androgen receptor (AR) expression 76. The proliferative effect of capsaicin on LNCaP cells was reversed by the TRPV1 antagonists 5-iodo-resiniferatoxin (I-RTX), capsazepine, and SB366791 76. In a similar study, Huang et al. demonstrated that capsaicin treatment promoted the proliferation of human esophageal squamous cell carcinoma (ESCC) Eca109 cells, and the effect was abolished by the TRPV1 receptor antagonist AMG9810 77. The information detailed in Table 2 indicates that the effect of capsaicin on proliferation is dependent on its concentration: low-dose capsaicin promotes cancer cell proliferation, whereas high-dose capsaicin prevents proliferation.

### Some agonists affect cell proliferation via both TRPV1 and other receptors

Some pharmacological regulators activate not only TRPV1 but also other reporters to affect cancer cell proliferation. For instance, cannabidiol prevented the proliferation of human breast cancer MBA-MD-231 cells through the activation of cannabinoid receptor type 2 (CB2) and TRPV1 to elevate intracellular Ca^2+^ and ROS generation and induce apoptosis 78. The respective CB2 and TRPV1 antagonists SR144528 and I-RTX partially rescued the antiproliferation effect of cannabidiol 78. Similarly, Aviello et al. suggested that cannabidiol reduces the phosphorylation levels of Akt to prevent human colon adenocarcinoma Caco-2 cells growth. This effect was counteracted by the CB1 receptor antagonists rimonabant and AM251 and the TRPV1 receptor antagonist capsazepine 79. Noradrenaline has been shown to induce Ca^2+^ flux by activating alpha 1D-AR and TRPV1 and, subsequently, eliciting the extracellular signal-regulated kinase 1/2 (ERK1/2), PLC, and PKC pathways to promote the proliferation of human prostate tumor PC-3 cells. This effect was completely reversed by alpha 1D-AR/TRPV1 double-knockdown or treatment with a combination of clopenphendioxan and capsazepine 80.

### TRPV1 agonists affect cell proliferation independently of TRPV1

Several research groups have reported TRPV1 agonists affecting cell proliferation in a TRPV1-independent manner. These agonists perform proliferation functions through two mechanisms. Firstly, the chemicals contribute to antiproliferation; for example, in the study carried out by Zhang et al., capsaicin induced apoptosis by increasing ERK phosphorylation and alleviating STAT3 phosphorylation, thus inhibiting the proliferation of a hepatocellular carcinoma cell line (PLC/PRF/5) cells. This was independent of TRPV1 81 because no TRPV1 expression was detected in PLC/PRF/5 or the other hepatocellular carcinoma cell lines (HuH7 and HepG2) 81. Capsaicin suppressed prostate cancer androgen-resistant PC-3 cell proliferation by inducing apoptosis via the production of ROS by the mitochondria and a decrease in perturbations in the inner transmembrane potential (△Ψm), which could not be reversed by capsazepine treatment, indicating TRPV1 was not involved 82. Capsaicin reduced the proliferation of human pancreatic neuroendocrine tumor BON and QGP-1 cells by disrupting the mitochondrial membrane potential and suppressing ATP synthesis to induce apoptosis, which was unaffected by a reduction in TRPV1 expression levels 83. Recently, we found that capsaicin inhibited cell proliferation in nasopharyngeal carcinoma (NPC) CNE2 and SUNE1 cells by directly targeting p38, leading to MKK3-p38 axis blockage, in a TRPV1-independent manner 84.

Secondly, the chemicals play vital roles in proliferation by activating receptors other rather than TRPV1. For example, cannabigerol, an agonist of TRPV1, prevented human Caco-2 cells growth by increasing CHOP mRNA levels and ROS production to stimulate apoptosis 85. This effect was alleviated in TRPM8-knockdown cells but not in cells treated with the TRPV1 antagonist ruthenium red 85. N-arachidonoylethanolamine (anandamide, AEA) inhibited the proliferation of murine neuroblastoma N1E-115 cells, and proliferation was rescued by a lipid raft disruptor, methyl-b-cyclodextrin, but not by the TRPV1 antagonist capsazepine 86.

## TRPV1 regulates cell death

Studies have demonstrated that pathological changes in or the pharmacological regulation of TRPV1 expression levels affect cell death. As mentioned in the previous section, TRPV1 overexpression or agonist treatment induces apoptosis, preventing cancer cell proliferation. Therefore, in this section, we focus on the roles of TRPV1 in cell death and provide a summary of the information (Table 3).

### TRPV1 expression affects cell death

In 2018, Yang et al. published a paper in which they described the overexpression of TRPV1 inducing a pro-apoptotic effect mediated by p53 activation 71. In 2016, Pecze and co-workers demonstrated that the ectopic expression of TRPV1 in human breast cancer MCF-7 cells led to apoptosis 87. The overexpression of fibulin‐5, a multifunctional extracellular matrix (ECM) protein encoded by the *FBLN5* gene, induced apoptosis in human colorectal cancer HT‐29 and SW480 cells by enhancing the phosphorylation of p38 and ERK and alleviating the level of p‐Akt by downregulating TRPV1 88. These studies indicate that TRPV1 can promote or inhibit cell death in a cancer- or tissue-specific manner.

### TRPV1 agonists affect cell death dependent on TRPV1

Apoptotic or necrotic cell death can be triggered by calcium influx 89. A large body of evidence shows the Ca^2+^-channel-activating function of TRPV1 evoked by its agonists is responsible for these chemically induced cell deaths. The overexpression of TRPV1 in human breast cancer MCF-7 cells has been reported to have no effect on cell numbers; however, the increased expression of exogenous TRPV1 meant that necrosis was more effectively induced by the capsaicin activation of TRPV1 and the subsequent upregulation of *c-Fos* and the necrotic marker *RIP3* 90. Arachidonyl ethanolamide induced apoptosis in human uterine cervix cancer C299, Caski, and HeLa cells, and this was reversed by capsazepine 91. Capsaicin induced apoptosis in human osteosarcoma G292 cells by activating endoplasmic reticulum TRPV1, which led to cytochrome C release 92. TRPV1 agonists, capsaicin and MRS1477, stimulated ROS production and mitochondrial membrane depolarization to induce apoptosis, which was blocked by capsazepine 93. In human endometrial cancer Ishikawa cells, AEA and cannabidiol caused apoptosis by activating TRPV1 to increase intracellular Ca^2+^ levels 94. Capsaicin also induced apoptotic and necrotic cell death in human breast cancer SUM149PT cells, and the reduction in cell viability mediated by capsaicin was diminished by capsazepine treatment 68. Taken together, it appears agonists of TRPV1, such as capsaicin, arachidonyl ethanolamide, MRS1477, AEA and cannabidiol, induce cell death by activating TRPV1 channels.

### TRPV1 agonists and antagonists affect cell death independently of TRPV1

In contrast, several studies have revealed that certain TRPV1 pharmacological regulators trigger cell death independently of TRPV1. For example, the induction of apoptosis in human cutaneous melanoma A375 cells by the TRPV1-activator AEA was completely counteracted by methyl-β-cyclodextrin, a membrane cholesterol depletory, but not by capsazepine 95. A similar effect was observed in non-melanoma skin cancer and colorectal cancer 96. Capsaicin treatment induced apoptosis in methylcholanthrene-induced fibrosarcoma Meth A cells, and this could not be inhibited by the antagonists capsazepine and I-RTX 97. Capsaicin induced apoptosis in human small cell lung cancer H69, DMS 114, DMS 53, and H82 cells by activating TRPV6 and, subsequently, inducing calpain-1 and calpain-2 activity. However, this pro-apoptotic effect was not abolished by the TRPV1 antagonist SB366791 98. Another report showed capsaicin-induced apoptosis in gastric cancer AGS cells was mediated by TRPV6 rather than TRPV1 99. Our recent results also indicate that capsaicin promoted apoptosis in NPC CNE2 and SUNE1 cells by inhibiting MKK3-induced p38 activation in a TRPV1-independent manner 84. R(+)-methanandamide (MA) treatment increased COX-2 and PPARγ activity and induced the apoptosis of human cervical carcinoma HeLa cells, an effect that was unaltered by capsazepine 100. The promotion of necrosis by resiniferatoxin was associated with mitochondrial dysfunction and was not reversed by I-RTX 101. Furthermore, Gonzales et al. illustrated that both capsaicin and capsazepine induce cell death independently of TRPV1 in oral squamous cell carcinoma HSC3, SCC4, and SCC25 cells 102.

## TRPV1 regulates cancer metastasis

Metastasis, which is one of the major causes of cancer-related deaths, requires two early events: migration and invasion 103. Recently, calcium signaling was found to be closely related to carcinogenesis and metastasis 104-106, yet only a few reports mention the role of TRPV1 in cancer cell metastasis. As TRPV1 serves as the main Ca^2+^-influx channel, it is reasonable to suggest that TRPV1 could act as an enhancer or inhibitor of migration and invasion in a tissue- or cell-specific manner.

### TRPV1 agonists and antagonists affect cell migration dependent on TRPV1

Few published reports describe the direct impact of altering TRPV1 expression on cancer cell metastasis. Most of the studies using agonists or antagonists showed an ambiguous effect of TRPV1 channel activity on migration. On the one hand, Vriens et al. and Waning et al. reported that the activation of TRPV1 can be evoked by hepatocyte growth factor (HGF) and causes the influx of Ca^2+^ into HepG2 cells following capsaicin treatment, leading to increased cell migration 107, 108. Another investigation into the pro-migration role of TRPV1 conducted on lymphatic endothelial cells by Nakanishi et al. found TRPV1 functioned as a pH sensor and was activated by an acidic environment, upregulating the expression of IL-8, and therefore promoting cell migration and invasion 109. On the other hand, Ramer et al. reported the activation of TRPV1 by MA did not affect cervix adenocarcinoma (HeLa) cell migration but inhibited cell invasion 110. In addition, a recent study by Xu et al. focusing on the function of capsaicin in papillary thyroid carcinoma BCPAP cells showed that capsaicin inhibited cell migration in a TRPV1-dependent manner 111. It is interesting that the activation of TRPV1 can lead to both cell death and increased migration. One possible explanation is that the concentrations of most of the agonists used in the pro-apoptosis/necrosis studies were at micromolar levels, and the treatment duration was usually 24 hours. In contrast, the concentrations of agonists used in the pro-migration studies were at nanomolar levels, or the treatment was provided over a short period (Tables 3 and 4). Although the exact mechanism is unclear, one suggestion is that the treatments differentially altered the levels of Ca^2+^ influx, which led to opposing effects.

### TRPV1 affects cell invasion

Unlike its effect on migration, the effect of TRPV1 on cancer cell invasion has been relatively well characterized and studied, and TRPV1 is believed to function as an invasion repressor. In 2005, Lazzeri et al. showed TRPV1 protein expression levels in urothelial cancer (UC) progressively decreased over progressive cancer metastasis stages 112. In the same vein, Kalogris et al. found this reduction to be not only translational but also transcriptional in UC. They also showed TRPV1 expression was positively related to survival rate, suggesting TRPV1 could be used as a factor for estimating the prognosis 113.

Evidence suggests TRPV1 may regulate the ECM, which plays a critical role in cell metastasis 12, 13 and, thus, the regulation of cell invasiveness. Ramer et al. demonstrated that the TRPV1 agonist MA can inhibit cell invasion in HeLa cells by inducing the expression of tissue inhibitor of MMPs (TIMP)-1 and, thereby, downregulating MMP2, in a TRPV1 dependent manner 110. In a later study, the same group found that intercellular adhesion molecule-1 (ICAM1) was upregulated by cannabidiol-elicited TRPV1-activation-mediated p42/44 activation in lung cancer cell lines A549, H358, and H460, subsequently inhibiting cell invasion 114. Additionally, Xu et al. showed capsaicin-elicited TRPV1 activation downregulated Twist1, Snail1, MMP2, and MMP9 expression and upregulated E-cadherin expression, thereby inhibiting cell invasion by papillary thyroid carcinoma BCPAP cells 111.

### TRPV1 agonists and antagonists affect cell migration independently of TRPV1

Investigations into TRPV1 commonly focus on the effects of treatment with agonists, e.g., capsaicin and cannabinoids, or antagonists such as capsazepine and AM404. However, the effects on metastasis either from TRPV1 activity alteration or the actions of agonists and antagonists should be interpreted with caution because of the reports of TRPV1 agonists and antagonists regulating cell migration and invasion in a TRPV1-independent manner. For instance, Caprodossi et al. found capsaicin promoted the more aggressive expression of genes, such as MMP1, MMP9, and S100A, and the invasiveness of null-*TRPV1* urothelial cancer 5637 cells 115. Whereas, in NPC, we found capsaicin inhibited cell mobility by blocking MKK3-induced p38 activation in a TRPV1-independent manner 84. Caballero et al. reported AM404, an antagonist of TRPV1 and CB1, inhibited NFAT transcriptional activity and, thus, downregulated MMP1, MMP2, and MMP7 in neuroblastoma SK-N-SH cells via a mechanism unrelated to TRPV1 116. Furthermore, a recent investigation conducted by Punzo et al. reported JWH133 and resiniferatoxin enhanced the antiproliferation, anti-invasion, and apoptosis effects of bortezomib (BTX), a selective, reversible proteasome inhibitor, in osteosarcoma cells. However, this report does not include direct evidence to show if the synergism was exerted by BTX-mediated TRPV1 upregulation or if the TRPV1 upregulation was an unrelated effect of BTX 117.

## TRPV1 affects cancer therapy

### TRPV1 agonists and antagonists affect cancer therapy dependent on TRPV1

To date, there has been some evidence associating TRPV1 expression with the efficiency of radiotherapy and chemotherapy, and several investigators have published the impact of TRPV1 agonists and antagonists on these treatments. In 2016, Nishino et al. reported that pretreatment with the TRPV1 channel inhibitors capsazepine, SB366791, AMG9810, and BCTC suppressed repair of γ-ray-induced-DNA-damage in human lung cancer A549 cells, indicating TRPV1 antagonists may serve as radiosensitizers, enhancing the efficacy of radiation therapy 118. Although there is no strong evidence that these chemicals impact the effectiveness of radiotherapy through TRPV1, Masumoto et al. demonstrated that depletion of TRPV1 suppressed the degree of DNA damage induced by γ-irradiation and UVB irradiation 119. Therefore, it is likely TRPV1 is involved in DNA-damage responses induced by radiotherapy. However, not all the effects and mechanisms have been clarified.

There are many pharmacological regulators of TRPV1, and several studies have focused on the roles of TRPV1 agonists/antagonists in chemotherapy (Table 5). The toxicity of cisplatin on breast cancer MCF-7 cells was found to be increased by TRPV1-channel activation by alpha-lipoic acid (ALA), the effect of which was reversed by the TRPV1 blocker capsazepine 120. Capsaicin increased the antiproliferative effects of pirarubicin, a major drug used in urinary bladder instillation chemotherapy, and the effect was reversed by capsazepine treatment 121. In human breast cancer MCF-7 cells, doxorubicin treatment activated TRPV1, resulting in increased intracellular Ca^2+^, which was reversed by the TRPV1 antagonist melatonin. Furthermore, a combination of doxorubicin and melatonin treatment led to higher apoptosis levels than doxorubicin treatment 122. TRPV1 was also activated by 5-fluorouracil, inducing apoptosis; however, 5-fluorouracil toxicity was downregulated by the TRPV1-channel inhibitor *Hypericum perforatum* in breast cancer MCF-7 cells 123. The TRPV1 agonist resiniferatoxin enhanced the antiproliferation effect of BTX in human osteosarcoma HOS cell lines by aggravating apoptosis 117. The above-described cases indicate that TRPV1 agonists and chemotherapeutic agents may have synergic effects in cancer therapy. Nevertheless, the roles of TRPV1 antagonists in cancer therapy are controversial.

### TRPV1 agonists and antagonists affect therapy independently of TRPV1

Several reports show that TRPV1 agonists/antagonists alter the sensitivity of chemotherapeutic agents without stimulating TRPV1. For instance, sorafenib is the standard systemic chemotherapy drug for the treatment of advanced hepatocellular carcinoma 124; capsaicin enhanced the antitumor sensitivity of sorafenib in hepatocellular carcinoma PLC/PRF/5 cells without obvious TRPV1 expression 81. Capsazepine amplified the antitumor activity of tumor necrosis factor-related apoptosis-inducing ligand (TRAIL) through multiple mechanisms, including promoting the expression of the death receptors DR4 and DR5 via the ROS-JNK-CHOP pathway; downregulating the expression of cell survival proteins cFLIP, survivin, Bcl-xL, Bcl-2, and cIAP-1; and upregulating the expression of proapoptotic proteins Bax and p53 125. However, these effects did not involve TRPV1 125.

## TRPV1 regulates tumor microenvironment

Tumors are not just a simple collection of cancerous cells, rather they are a complex of tumor cells and their interactions with the surrounding cells, forming the tumor microenvironment (TME). Recent reviews have indicated TME is an important mediator in cancer progression and is mainly composed of (a) ECM, which provides structural and nutritional support for tumor development; (b) tumor vasculature, which carries oxygen and nutrients to tumor cells and a provides a “highway” for metastasis; (c) cancer-associated fibroblasts, which contribute to tumor proliferation and metastasis, as well as regulating the formation of ECM; and (d) the immune system, which plays a critical part in tumor-related inflammation 126-128. The contribution of the important intercellular and intracellular messengers, Ca^2+^ ions, to the TME has been reviewed elsewhere 105, 129-131. As TRPV1 is a major Ca^2+^ channel, we summarize the relationships between TRPV1 and TME in the following section.

### TRPV1 and cancer-associated fibroblasts

The final outcome of cancer development is not only determined by autonomous cancer cell defects but also by interactions between cancer cells and the TME. Cancer-associated fibroblasts are major components of the tumor stroma, and evidence collated over many years indicates that fibroblasts are key players in cancer development 132, 133. Cancer-associated fibroblasts (CAF) can be recruited and activated by cancer-secreted growth factors, among which TGF-β is the main factor contributing to their activation 134-136.

Associations between TRPV1 and TGF-β in somatic cells have been reported for many years. For instance, Bodo et al. demonstrated that the activation of TRPV1 by capsaicin upregulated TGF-β2 mRNA and protein expression in human hair follicles through an unknown mechanism 137. This was supported by further studies, which showed expression of TGF-β1 was attenuated in a TRPV1-knock-out animal model 138-140. These findings hint at the possible regulation of TGF-β by TRPV1, yet currently, there is no evidence for correlation or interaction between TRPV1 and TGF-β in cancer. As fibroblasts act as important mediators for other TME components 132, 133, it would be interesting to further investigate the relationship between TRPV1 and TGF-β in cancer cells and cancer-related fibroblasts.

### TRPV1 and ECM

ECM is mainly composed of the basement membrane, which primarily comprises collagen, and the interstitial matrix containing proteoglycans and fibrous proteins 141. Hence, TRPV1 is a transmembrane protein that functions as a temperature and pH sensor and a non-selective cation channel; it is unsurprising, therefore, that the ECM and TRPV1 are closely linked. According to previous research, several components of the ECM are able to modulate TRPV1 activity; for instance, extracellular hyaluronan (hyaluronic acid) reduced the excitation of the TRPV1 channel, thereby reducing the activity of peripheral nociceptors 142. Moreover, another ECM protein, fibulin-5, was reported to induce apoptosis in colorectal cancer cells by downregulating TRPV1 expression 88. However, TRPV1 is also capable of regulating ECM proteins, including the main regulators of collagen in ECM matrix metalloproteinases (MMPs), which are highly relevant to the metastatic processes of cancer cells 143, 144. In the immortalized somatic cell, TRPV1 was reported to directly mediate the expression of MMP-1 145, 146, and Huang et al. further elucidated that TRPV1 regulates MMP1 expression via the Ca^2+^-ERK pathway 147. In the field of oncology, there have been several reports showing that TRPV1 activation in cancer cells regulates MMP activity by modulating the expression of TIMP-1 110, 148. However, there were no details of the mechanisms and signaling pathways involved in the TRPV1-regulation of TIMP-1 expression, and these should be investigated in the future. Moreover, whether TRPV1 regulates other ECM proteins is unclear.

### TRPV1 and cancer-associated angiogenesis

During angiogenesis, new blood vessels emerge from the existing vasculature via sprouting, which is vital for oxygen and nutrient transportation and is implicated in cancer metastasis. As early as 1995, the first links between the regulation of angiogenesis and calcium signaling were discovered by Kohn et al., who reported that carboxyamidotriazole (CAI), a non-voltage-operated Ca^2+^-channel inhibitor, inhibited vascular tube formation on Matrigel and angiogenesis *in vivo* 149. Faehling et al. further suggested that angiogenesis requires Ca^2+^ influx; vascular endothelial growth factor (VEGF) induced Ca^2+^ influx into human umbilical vein endothelial cells (HUVEC), and adding CAI inhibited the VEGF-elicited Ca^2+^ influx, inhibiting endothelial cell proliferation 150. There are several detailed reviews available covering Ca^2+^ signaling and angiogenesis, which are not elaborated on here 151-153.

The process of angiogenesis requires the proliferation and motility of endothelial cells, in which several critical proteins, i.e., VEGF, EGFR, and fibroblast growth factor, play important roles 132, 154-156. Among these, VEGF is considered the most potent factor for endothelial cell proliferation and migration. In the context of cancer, VEGF can be released from tumor cells or the tumor-related ECM and binds to VEGFR1/2 to induce the proliferation of vascular endothelial cells 157. As mentioned above, VEGF is able to induce Ca^2+^ influx into HUVECs 150. Indeed, VEGF has recently been reported to mediate Ca^2+^ influx by transactivating the channel function of TRPV1. In addition, VEGF secreted by human uveal melanoma cells transactivates TRPV1 function, causing intracellular Ca^2+^ influx into endothelial cells, which is essential for angiogenesis 158. Interestingly, Su et al. showed Ca^2+^ influx by simvastatin elicited TRPV1 activation of the AKT signal, which subsequently activated calcium-calmodulin kinase II (CaMKII) and eNOS, leading to angiogenesis 159. VEGF transactivation of TRPV1 might share a similar pathway, although more experimental data is needed to support this hypothesis.

Whether TRPV1 activity regulates the expression and function of VEGF and its receptor is still largely unknown; however, hints have been provided by animal models. Vinuesa et al. showed that *Trpv1* -/- mice were more vulnerable to dextran-sodium-sulfate-induced colon cancer 160. Their investigation demonstrated that the NF-kB and STAT3 signal pathways were hyperactivated in *Trpv1* -/- mice, resulting in the upregulation of a group of inflammatory factors, including IL-1 and IL-6, and invasion factors such as MMP9, which subsequently enhanced the carcinogenesis 160. NF-kB and STAT3 are known to be regulators of VEGF in several cancers 161-163 and may be involved in the regulation of VEGF expression by TRPV1.

### TRPV1 in inflammation and leukocytes

A considerable fraction of TME components is associated with inflammation and leukocyte activities. Cytokines released from tumor cells and TME components attract immune cells and trigger inflammation. Several systematic reviews have pointed out that inflammation contributes to tumorigenesis and helps to shape tumor progression, and modulating immune factors in the TME is a promising therapeutic approach for cancer treatment 164-166. The characteristics of inflammation include heat and pain, and, because TRPV1 functions as a nociceptor, it may be highly relevant to this area of research.

TRPV1 is involved in key immune cell functions, and Ca^2+^ signaling is important for lymphocyte activation and differentiation 167, 168. The functional expression of TRPV1 in mouse and human CD4+ T-cells was described by Bertin et al., who showed NFAT and NFκB, two key transcription factors of immune cell activation, were less expressed in the nuclear fraction of CD4+ T-cells isolated from *Trpv1* knockout mice. This resulted in the reduced production of proinflammatory cytokines and indicated that TRPV1 is necessary for proper downstream T-cell signaling 169. Moreover, TRPV1 was also shown to be expressed in dendritic cells (DCs), which are pivotal in antigen presentation and lymphocyte activation. Notably, the administration of capsaicin induced the maturation of DCs in *Trpv1* +/+ mice but not in *Trpv1* -/- mice 170.

TRPV1 was also reported to be a modulator for inflammatory cytokines, although the exact regulatory network for TRPV1-inflammation is unclear. Okada et al. showed the expression of several pro-inflammation cytokines, such as MCP-1 and IL-6, was suppressed during the healing of eye injury in *Trpv1* -/- mice, and they suggested that TRPV1 may serve a pro-inflammatory role 138. However, the story is completely reversed in cancer. In the AOM/DSS-induced colon cancer mouse model, several pro-inflammation factors, including IL-6 and IL-11, were found to be upregulated 160. Further research demonstrated that *Trpv1*-deficient mice exhibited hyperactivation of the STAT and NFκB signal pathways; therefore, TRPV1 was believed to exert a protective role in colon cancer 160. The findings of a recent study by Erin were in line with this view, as they showed the activation of TRPV1 by capsaicin downregulated the expression of TNF-a, IL-6, and IL-10 and suppressed lung metastasis in a breast cancer metastasis mouse model 171. However, something that should be brought to the reader's attention is that this study did not rule out the possibility that capsaicin functioned independently of TRPV1. Furthermore, because the above results were based on a TRPV1-deficient animal model, the regulation of inflammatory factors was more likely to be an outcome of systematic changes. Whether TRPV1 regulates the secretion of inflammatory cytokines by cancer cells is not fully understood, and the mechanisms need to be further investigated.

## Conclusion

Based on the reviewed evidence, the following conclusions can be drawn. The expression of TRPV1 is elevated in many cancers, and its overexpression suppresses cell proliferation in intestinal, melanoma, and pancreatic cancers and induces apoptosis in melanoma and breast cancers. These findings support the hypothesis that TRPV1 is a tumor-suppressor gene. However, TRPV1 either promotes or inhibits cell death, depending on cancer cell type, implying it has cancer- or tissue-specific functions. Further investigations and experimentations are strongly recommended.

From the current clinical and experimental data, it is likely that TRPV1 serves as a repressor of metastasis, which is in line with its tumor-suppressing role. Whereas the data showing TRPV1 to be a pro-metastasis factor suggest the role of TRPV1 in metastasis may be context-dependent.

As we mentioned, numerous studies have revealed that TRPV1 agonists/antagonists affect cancer proliferation, cell death, and metastasis by activating TRPV1 channels and subsequently increasing the levels of intracellular Ca^2+^. However, the effects are complex and are determined by the concentration used and treatment duration. Additionally, agonists or antagonists may activate other receptors and, therefore, function in a TRPV1-independent manner. Thus, the effects and mechanisms are unclear, and further studies using combined gene silencing or knockout approaches are needed.

**Figure 1:**
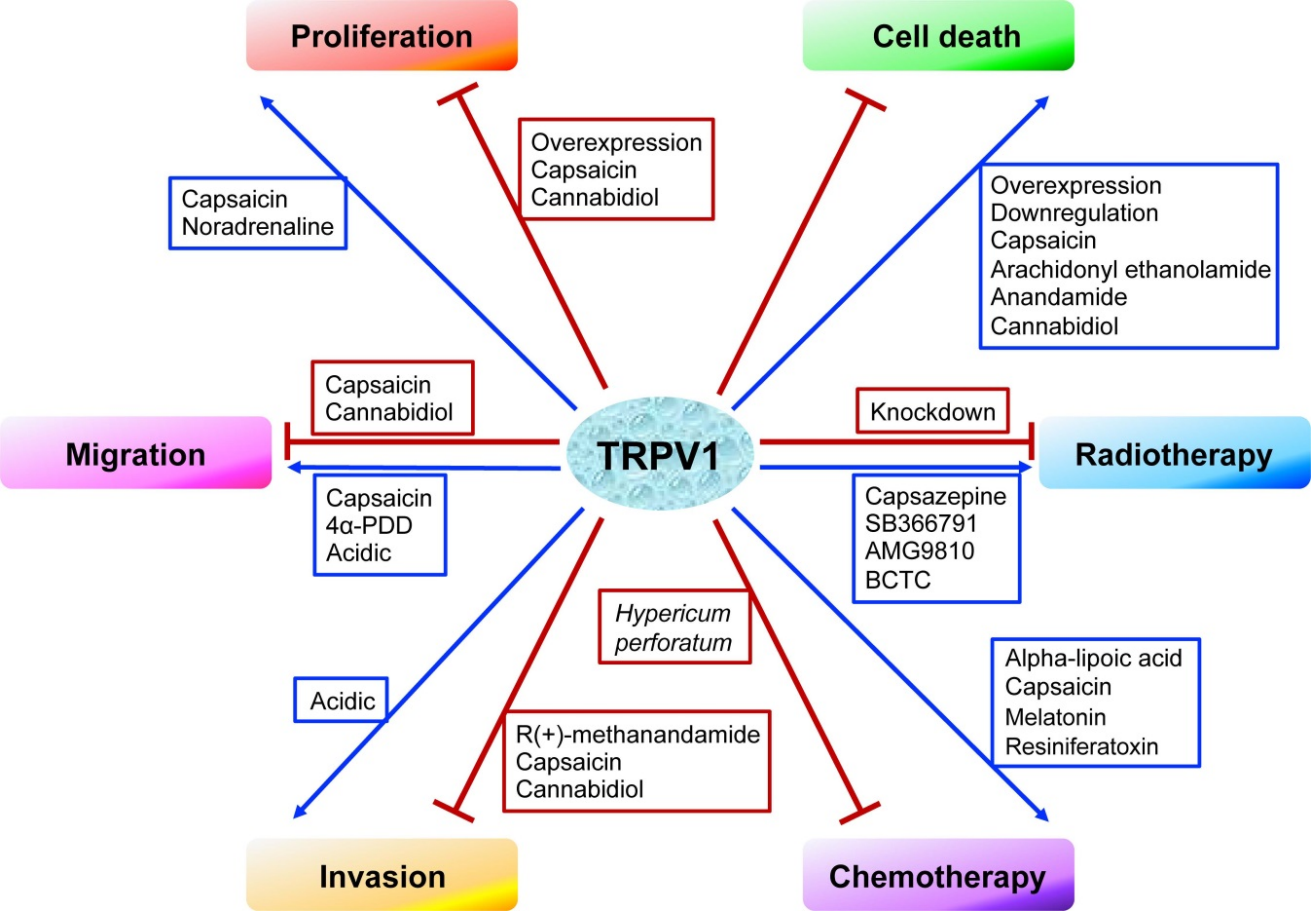
The effects of TRPV1 on cancer cell proliferation, cell death, migration, invasion, radiotherapy, and chemotherapy.

**Figure 2:**
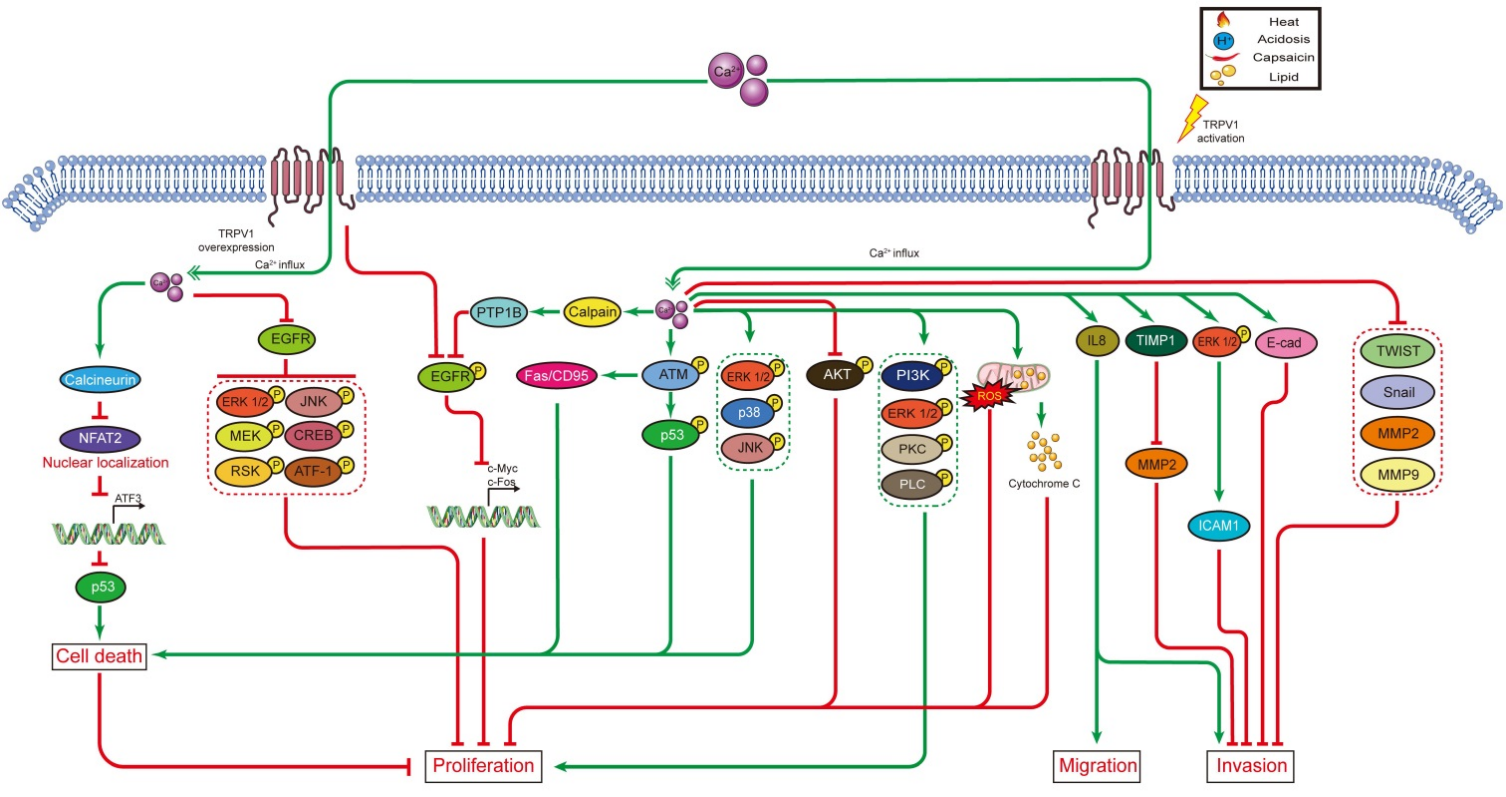
TRPV1 signaling pathways in cancer cell proliferation, cell death, migration, and invasion.

**Figure 3:**
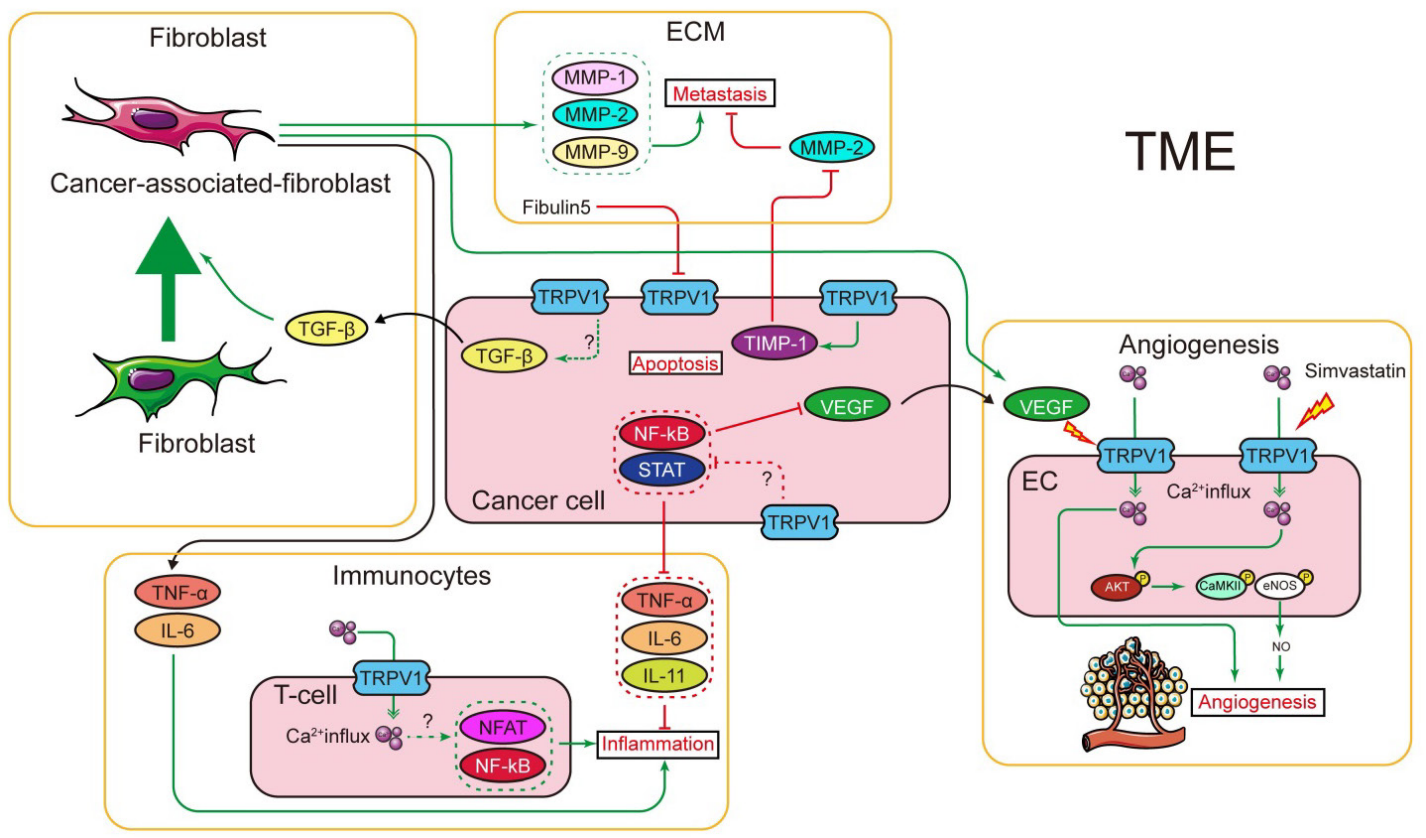
Interaction between TRPV1 and tumor microenvironment. Using transgenic mice, TRPV1 was shown to regulate TGF-β, which is the major factor that activates fibroblasts and transforms the fibroblasts into CAF. The CAF can secret several cytokines and factors, most of which are TME components, e.g., MMPs (regulates collagen in ECM), TNF-α and IL-6 (trigger inflammation), and VEGF (promotes angiogenesis). In cancer cells, TRPV1 was shown to regulate the expression of several inflammation factors, probably through NFκB and STAT signaling pathways. In immunocytes, TRPV1 is important for T-cell signal transduction. Additionally, TRPV1 can regulate the ECM by promoting TIMP-1 expression. In turn, fibulin 5, an ECM component, downregulates TRPV1 expression in cancer cells. Furthermore, TRPV1 was shown to promote angiogenesis by mediating Ca^2+^ influx and, subsequently, activating the AKT-CaMKII-eNOS signal pathway. The TRPV1 channel function can also be elicited by VEGF, but it is unclear if it shares the same downstream signal.

In cancer chemotherapy, the TRPV1 agonist capsaicin has a synergic effect with cisplatin. Nevertheless, capsaicin cannot be systemically administered in large doses, as it induces acute pain and neurological inflammation. In short, current evidence indicates TRPV1 plays certain roles in shaping the TME. However, the signaling networks interacting with TRPV1 have not been fully characterized, and the relationships between TRPV1 and TME need to be further explored. The effects of TRPV1 on cancer cell proliferation, cell death, migration, and invasion, as well as on radiotherapy and chemotherapy, are summarized in Figure 1. Moreover, the TRPV1 signaling pathways in cancer cell proliferation, cell death, migration, and invasion described in this review are summarized in Figure 2. The association between TRPV1 and TME are summarized in Figure 3.

Finally, a number of important issues need to be considered. Given the fact that TRPV1 also plays several intracellular roles, it remains unclear whether TRPV1 functions independently of its channel activity in cancer progression. Further research with more focus on the role of the *TRPV1* gene in tumorigenesis and development using gene overexpression and knockdown (knockout) approaches is therefore suggested.

## Figures and Tables

**Table 1:** Endogenous and exogenous ligands of TRPV1.

Ligands	Ref.
Endogenous agonists:	
Anandamide	25
N-arachidonoyldopamine	26
N-oleoyldopamine	27
R(+)-methanandamide	25
12- and 15-hydroperoxyeicosatetraenoic acid	28
5- and 15-hydroxyeicosatetraenoic acid	28
Leukotriene B4	28
9- and 13-hydroxy-octadecadienoic acid	12
9- and 13-oxo-octadecadienoic acid	12
Oleoylethanolamide	29
Palmitoylethanolamide	30
Lysophosphatidic acid	31
Oxytocin	32
Exogenous agonists	
2-aminoethoxydiphenyl borate	33
4α-phorbol-12,13-didecanoate	34
*Ornithoctonus huwena* toxin ['double-knot' toxin (DkTx)]	35
Capsaicin	8
Piperine	36
Resiniferatoxin	37
Gingerol	38
Evodiamine	39
Cannabidiol	40
Cannabigerol	41
Polygodial	42
Vanillotoxin	43
MD-652	44
Linopirdine	45
Endogenous antagonists	
Resolvin D2	46
Noradrenaline	47
Exogenous antagonists	
Capsazepine	48
Iodo-resiniferatoxin	49
Hypericum perforatum	50
JNJ-17203212	51
BCTC	52
Thapsigargin	53
Yohimbine	54
JYL 1421	55
Caffeic acid	56
Asivatrep	57
SB-366791	58
A-1165442	59
AMG 9810	60
AG489, AG505	61
ABT-102, AMG-517, AZD-1386, DWP-05195, GRC-6211, JTS-653, MK-2295, PHE377, SB-705498	62, 63

**Table 2:** Role of TRPV1 in Proliferation.

Drug	Dose (μM)	Duration (h)	Tissue/Cell Type	Mechanism	Outcomes	Ref.
			Human colorectal cancer HCT116 cells	Overexpression of TRPV1 suppressed EGFR phosphorylation at Y1068	Proliferation ↓	70
			*Trpv1* knockout mice	*TRPV1* knockout increased constitutive EGFR Y1068 phosphorylation and PCNA, *c-Fos* and *c-Myc* expression levels	Proliferation ↑	70
			Human colorectal cancer HCT116 cells	TRPV1 activation activates calpain and PTP1B, which dephosphorylates EGFR	Proliferation ↓	70
			Human melanoma A2058 and A375 cells	Overexpression of TRPV1 activated p53 and, subsequently, upregulated its downstream target genes p21, PUMA, and mdm2 to induce apoptosis	Proliferation ↓	71
			Human pancreatic cancer cell line, PANC-1	Overexpression of TRPV1 downregulates EGFR levels by inducing EGFR ubiquitination and degradation	Proliferation ↓	72
			Human skin A431 cells	Overexpression of TRPV1 promotes EGFR ubiquitylation and lysosomal degradation	Proliferation ↓	73
Capsaicin	100	24	Human urothelial cancer RT4 cells	Induced cell cycle arrest in G0/G1 phase and apoptosis by activating p53 to upregulate Fas/CD95 in TRPV1-overexpressing cells	Proliferation ↓	74
Capsaicin	50-400	24-48	Human renal carcinoma 786-O cells	Activated p38 and JNK MAPK pathways to induce apoptosis	Proliferation ↓	75
Capsaicin	0.1-20	48	Human prostate tumor androgen-responsive LNCaP cells	Activated PI3K and p44/42 MAPK pathways to suppress ceramide production and increased androgen receptor expression	Proliferation ↑	76
Capsaicin	15	96-120	Human ESCC cell lines Eca109		Proliferation ↑	77
Capsaicin	100	24	Human hepatocellular carcinoma PLC/PRF/5 cells	Induced apoptosis by increasing the phosphorylation level of ERK and attenuating STAT3 phosphorylation	Proliferation ↓	81
Capsaicin	20	36	Human prostate tumor androgen-resistant PC-3 cells	Induced apoptosis by producing ROS originating from the mitochondria and decreasing perturbations in the inner transmembrane potential (△Ψm) independently of TRPV1	Proliferation ↓	82
Capsaicin	100	24-72	Human pancreatic neuroendocrine tumor BON and QGP-1 cells	Disrupted mitochondrial membrane potential and suppressed ATP synthesis to induce apoptosis	Proliferation ↓	83
Capsaicin	75	24/48	Human nasopharyngeal carcinoma CNE2 and SUNE1 cells	Inhibited MKK3-induced p38 activation	Proliferation ↓	84
Capsaicin	3	48/96	Human breast carcinoma cell line MCF-7 cells		Proliferation ↑	90
Cannabidiol	10	48	Human breast carcinoma cell line MBA-MD-231 cells	Induced apoptosis via activation of CB2 and TRPV1 to elevate reactive oxygen species	Proliferation ↓	78
Cannabidiol	10	24	Human colon adenocarcinoma cell line Caco-2 cells	Reduced the phosphorylation level of Akt, which was dependent on TRPV1 and CB1	Proliferation ↓	79
Noradrenaline	100	24	Human prostate tumor androgen-resistant PC-3 cells	Activated both alpha 1D-AR and TRPV1 and, subsequently, elicited the PLC/PKC/ERK pathways	Proliferation ↑	80
Cannabigerol	10	24	Human colon adenocarcinoma cell line Caco-2 cells	Stimulated ROS generation, increased CHOP expression level, and promoted apoptosis	Proliferation ↓	85
AEA	10	24-72	The murine neuroblastoma cell line N1E-115	Seemed to occur via a lipid raft-dependent mechanism	Proliferation ↓	86

**Table 3:** Role of TRPV1 in Cell Death.

Drug	Dose (μM)	Duration (h)	Tissue/cell type	Mechanism	Outcomes	Ref.
Capsaicin	3	48/96	Human breast carcinoma cell line MCF-7 cells	Activated exogenous TRPV1 and, subsequently, upregulated *c-Fos* and necrotic marker *RIP3*	Necrosis	90
Capsaicin	150	24	Human osteosarcoma G292 cells	Activated endoplasmic reticulum TRPV1 and induced cytochrome C release	Apoptosis	92
Capsaicin	10	72	Human breast cancer MCF-7 cells	Stimulated ROS production and mitochondrial membrane depolarization	Apoptosis	93
Capsaicin	150	48	Human breast cancer SUM149PT cells	Activated TRPV1	Apoptosis Necrosis	68
Capsaicin	100	72	Human Methylcholanthrene-induced fibrosarcoma Meth A cells	Decreased Fas-associated factor1 (FAF1) expression level	Apoptosis	97
Capsaicin	50	36	Human small cell lung cancer H69, DMS 114, DMS 53, and H82 cells	Activated TRPV6 and, subsequently, induced calpain-1 and calpain-2 activation	Apoptosis	98
Capsaicin	50	1/6/24	Human gastric cancer AGS cells	Disrupted mitochondrial integrity, activated JNK and, thus, led to TRPV6-mediated p53 stabilization	Apoptosis	99
Capsaicin	37.5/75	24/48	Human nasopharyngeal carcinoma CNE2 and SUNE1 cells	Inhibited p38 phosphorylation mediated by MKK3	Apoptosis	84
Capsaicin	150	24	Human oral squamous cell carcinoma HSC3, SCC4 and SCC25 cells		Cell death	102
Arachidonyl ethanolamide	30	48	Human uterine cervix cancer C299, Caski, and HeLa cells	Activated TRPV1	Apoptosis	91
Anandamide/ cannabidiol	50/25	48	Human endometrial cancer Ishikawa cells	Activated TRPV1 to increase intracellular calcium levels	Apoptosis	94
Anandamide	10	24	Human cutaneous melanoma A375 cells	A complex mechanism comprising CB1 activation, COX-2, and LOX-derived product synthesis	Apoptosis	95
Anandamide	20	4	The murine squamous carcinoma cell line JWF2 and human colorectal cancer cell line, HCA-7 Colony 29	Induced oxidative stress and ER-stress apoptosis	Apoptosis	96
MRS1477	2	72	Human breast cancer MCF-7 cells	Stimulated ROS production and mitochondrial membrane depolarization	Apoptosis	93
R(+)-methanandamide (MA)	10	24-72	Human cervical carcinoma HeLa cells	Increased COX-2 expression and activity	Apoptosis	100
Resiniferatoxin	20	24	Human bladder cancer T24 and 5637 cells	Induced mitochondrial dysfunction	Necrosis	101
Capsazepine	30	24	Human oral squamous cell carcinoma HSC3, SCC4 and SCC25 cells	Stimulated ROS production	Apoptosis	102

**Table 4:** Role of TRPV1 in Metastasis.

Drug	Dose	Duration	Tissue/cell type	Mechanism	Outcomes	Ref.
Capsaicin	10 μM	400 s	Hepatoblastoma HepG2 cell	HGF evoked TRPV1 channel activity, causing Ca^2+^ influx	Migration ↑	107
Capsaicin	100 nM	5 h	HGF pretreated hepatoblastoma HepG2 (20 ng/mL for 24 h) cells	HGF evoked TRPV1 channel activity, causing Ca^2+^ influx	Migration ↑	108
Capsaicin	25/50/100 μM	24/48 h	Human papillary thyroid carcinoma BCPAP cells	Capsaicin downregulated Tiwst1, Snail1, MMP2, and MMP9 and upregulated E-cadherin	Migration ↓ Invasion ↓	111
Capsaicin	100 μM	24 h	Urothelial cancer 5637 cells (null-TRPV1)	Capsaicin treatment induced more aggressive gene expression, e.g., MMP1, MMP9, and S100A4	invasion ↑	115
Capsaicin	25/37.5/75 μM	48 h	Human nasopharyngeal carcinoma CNE2 and SUNE1 cells	Capsaicin directly targeted p38 and blocked the MKK3-p38 axis	Migration ↓ Invasion ↓	84
4α-PDD	1 μM	5 h	HGF pretreated hepatoblastoma HepG2 ( 20 ng/mL for 24 h) cells	HGF evoked TRPV1 channel activity, causing Ca^2+^ influx	Migration ↑	108
			Primary human adult dermal LECs (HDLECs) incubated with pH-6.4 starvation medium	Acidic environment elicited TRPV1 activation, which upregulated IL-8 expression	Migration ↑	109
MA	10 μM	72 h	Cervix adenocarcinoma HeLa cells	MA elicited TRPV1 activation and upregulated TIMP-1 expression via MAPK pathway, causing decrease in MMP2	Invasion ↓	110
Cannabidiol	3 μM	72 h	Human lung cancer A549, H358, and H460 cells	CBD elicited TRPV1 activation, upregulated ICAM1 expression via phosphorylating p42/44, and subsequently upregulated TIMP-1	Migration ↓ Invasion ↓	114
AM404	5/15/25 μM	24 h	Neuroblastoma cells SK-N-SH	AM404 inhibited NFAT transcriptional activity, thus, downregulating MMP1, MMP2, and MMP7	Migration ↓ Invasion ↓	116
Bortezomib	10 nM	24 h	Osteosarcoma HOS cell	Inhibited TRPV1 degradation	Invasion ↓	117

**Table 5:** Role of TRPV1 in Cancer Therapy.

Drug	Dose (μM)	Duration (h)	Tissue/cell type	Mechanism	Outcomes	Ref.
Capsaicin	100	24	Human hepatocellular carcinoma PLC/PRF/5 cells	Induced apoptosis by increasing phosphorylation levels of ERK and attenuating STAT3 phosphorylation	Increased antitumor sensitivity of sorafenib	81
Capsaicin	150	12	Human bladder transitional cell carcinoma 5637 cells	Inhibited PCNA translocation to the nucleus	Increased antitumor efficacy of pirarubicin	121
Capsazepine	10	6	Human colorectal cancer HCT116 cells	Mediated induction of expression of death receptors DR4 and DR5 via ROS-JNK-CHOP pathway, downregulated the expression of cell survival proteins, and upregulated the expression of proapoptotic proteins	Increased antitumor activity of TRAIL	125
Capsazepine/SB366791/AMG9810/BCTC	10	0.5	Human lung cancer A549 cells		Enhanced cell death induced by γ-rays	118
Alpha-lipoic acid	50	24	Human breast carcinoma cell line MCF-7 cells	Induced apoptosis via activation of TRPV1	Increased antitumor sensitivity of cisplatin	120
Melatonin	300	2	Human breast carcinoma cell line MCF-7 cells	Increased apoptosis induced by doxorubicin	Increased antitumor sensitivity of doxorubicin	122
Hypericum perforatum	300	24	Human breast carcinoma cell line MCF-7 cells	Downregulated apoptosis induced by 5-fluorouracil	Decreased antitumor sensitivity of 5-fluorouracil	123

## Abbreviations

TRPV1: transient receptor potential cation channel subfamily V, member 1
TRP: transient receptor potential
VR1: vanilloid receptor 1
EGFR: epidermal growth factor receptor
PLC: phospholipase C
PIP_2_: phosphatidylinositol-4,5-bisphosphate
DAG: diacylglycerol
IP_3_: inositol triphosphate
PTP1B: protein tyrosine phosphatase 1B
AR: androgen receptor
I-RTX: 5-iodo-resiniferatoxin
ESCC: esophageal squamous cell carcinoma
ERK: extracellular signal-regulated kinase
NPC: nasopharyngeal carcinoma
AEA: anandamide
FAF1: Fas-associated factor1
ECM: extracellular matrix
MA: R(+)-methanandamide
HGF: hepatocyte growth factor
UC: urothelial cancer
ICAM1: intercellular adhesion molecule-1
BTX: bortezomib
ALA: alpha-lipoic acid
TRAIL: tumor necrosis factor-related apoptosis-inducing ligand
TME: tumor microenvironment
MMP: matrix metalloproteinase
CAF: Cancer associated fibroblasts
DC: dendritic cell
CAI: carboxyamidotriazole
VEGF: vascular endothelial growth factor
CaMKII: calcium-calmodulin kinase II
HUVEC: human umbilical vein endothelial cell
FGF: fibroblast growth factor
